# Extraction and Characterization of Alginate from an Edible Brown Seaweed (*Cystoseira barbata*) Harvested in the Romanian Black Sea

**DOI:** 10.3390/md17070405

**Published:** 2019-07-08

**Authors:** Bogdan Trica, Cédric Delattre, Fabrice Gros, Alina Violeta Ursu, Tanase Dobre, Gholamreza Djelveh, Philippe Michaud, Florin Oancea

**Affiliations:** 1 Department of Bioresources, National Institute for Research & Development in Chemistry and Petrochemistry-ICECHIM Bucharest, Splaiul Independenței 202, 060021 Bucharest, Romania; 2 CNRS, SIGMA Clermont, Institut Pascal, Université Clermont Auvergne, F-63000 Clermont-Ferrand, France; 3 Department of Chemical and Biochemical Engineering, University “POLITEHNICA” of Bucharest, Splaiul Independenței 313, 060042 Bucharest, Romania

**Keywords:** alginate, *Cystoseira barbata*, Black Sea, heavy metals adsorption, diffusion model

## Abstract

*Cystoseira barbata* is an edible brown seaweed, traditionally used in the Black Sea area as functional food. Both alginate and brown seaweed biomass are well known for their potential use as adsorbents for heavy metals. Alginate was extracted from *C. barbata* recovered from the Romanian coast on the Black Sea with a yield of 19 ± 1.5% (*w*/*w*). The structural data for the polysaccharide was obtained by HPSEC-MALS, ^1^H-NMR. The M/G ratio was determined to be 0.64 with a molecular weight of 126.6 kDa with an intrinsic viscosity of 406.2 mL/g. Alginate beads were used and their adsorption capacity with respect to Pb^2+^ and Cu^2+^ ions was determined. The adsorption kinetics of *C. barbata* dry biomass was evaluated and it was shown to have an adsorption capacity of 279.2 ± 7.5 mg/g with respect to Pb^2+^, and 69.3 ± 2 with respect to Cu^2+^. Alginate in the form of beads adsorbs a maximum of 454 ± 4.7 mg/g of Pb^2+^ ions and 107.3 ± 1.7 mg/g of Cu^2+^ ions.

## 1. Introduction

Edible seaweeds have significant potential as functional food, especially as they are active against various human non-communicable diseases such as cardiovascular diseases, cancers, type 2 diabetes/metabolic syndrome, auto-immune diseases, due to their high level of bioactive components—polysaccharides, peptides, polyunsaturated fatty acids, polyphenols, vitamins and minerals [1,2]. One of the main active components of edible brown seaweed is alginate, a polysaccharide composed of two different uronic acids, mannuronic and guluronic [3]. Due to the fact that alginates are not digested by human enzymes, they act as prebiotics, supporting the production of short-chain fatty acids, and as potential immunomodulators [4].

Traditionally, alginates have been used as tablet excipients and for the treatment of stomach ulcers, gastric reflux and heartburn [5]. Alginates’ absorptive, swelling and haemostatic features are involved in their mode of action against such health conditions. These features also substantiate the use of alginate in wound treatment [6]. Their absorptive and swelling features have also been linked to many other health-related effects. For example, binding of glucose and α-amylase inhibition, which reduce post-prandial glucose levels [7,8]. Alginates (calcium) have the ability to absorb bile acids and lipids and therefore to lower cholesterol [9] and lipid levels [10].

The adsorption of heavy metal ions (biosorption) from the intestinal system is related to organism detoxification. Treatment with calcium alginate in a dose of 500 mg/kg removes the lead accumulated in rats after intoxication with lead acetate [11]. Simultaneous intoxication of lead acetate and treatment with calcium alginate significantly reduced lead accumulation in rat organs [12]. Administration of alginate in combination with modified citrus pectin reduces the total body burden of heavy metals [13].

Heavy metals are highly toxic to the environment and to humans. The recommended values for human consumption are in the very low ppm ranges: Pb, 0.01 ppm; Cu, 2 ppm; Hg, 0.001 ppm; As, 0.01 ppm; Ni, 0.02 ppm; etc. [14]. High levels of heavy metals have been reported in sources of drinking water [15], which have been contaminated from a variety of sources such as pipe corrosion [16,17] or industrial activities [18,19,20]. Other activities that have been associated with high contamination risks are petrochemistry [21] and electronics waste disposal (E-waste) [22]. In many cases, the recovery of heavy metal contaminants using adsorbents [15,23] is mainly due to their reduced costs as compared to other methods such as membrane filtration, chemical precipitation, ion exchange and others [15,24,25,26].

Biosorption is the process by which some type of biological material is used as an adsorbent to bind certain compounds [27]. It is regarded as a promising alternative to classical methods due to its cost-effectiveness and environment-friendly nature [21]. The biosorbents that are currently under development are mostly trying to take advantage of the adsorption properties of natural biomasses [28], or of composite materials based on natural sources [21,29]. Apart from bacteria [30,31,32] and fungi [21,33,34], agricultural [35] and algae-based adsorbents [28,36] have also been used. Among macroalgae, brown seaweeds are of particular interest because they contain alginate, which has a chemical affinity for divalent metals [37,38]. In fact, alginate chains form a tridimensional matrix in the presence of divalent metals by ionic crosslinking following the egg-box model [39]. Ca^2+^ ions are used for this purpose. However, divalent metals of higher atomic mass replace the calcium ions as the active sites in the alginate hydrogel have increased affinity for larger divalent metal ions [40]. For decades, alginate has been extracted from brown seaweed with a good yield at an industrial level to be used as a thickening and gelling agent in the food and cosmetics industry [41,42]. The main species that are currently exploited industrially are *Macrocystis pyrifera*, *Laminaria hyperborea*, *Laminaria digitata* and *Asphyllum nodosum* [43]. However, alternative uses are proposed for alginate in the biomedical field as a drug delivery system, for wound healing and tissue engineering [6], or as a biosorbent material. In fact, both brown seaweed biomass and alginate, in the form of gel beads have been extensively studied as biosorbents [28,37,44,45,46,47]. Recently, composite materials based on alginate have been derived with improved properties. For instance, including magnetite has enhanced the recovery of biosorbents. In this case, adsorption of Cd^2+^ ions was improved by also including activated carbon in the alginate matrix leading to an improved material in terms of adsorption capacity and costs [48]. Another approach is to obtain a compressive alginate sponge where the contaminant is recovered after adsorption simply by compressing the biosorbent before reuse. The efficiency of this system was proven in the case of treating methylene blue contaminated wastewaters [49]. The crosslinked alginate matrix has also been used as a coating for composite materials that are to be used as controlled drug release systems [50].

*Cystoseira* is a polyphyletic genus of brown seaweed, included in the Sargassaceae family, which is found extensively on the coasts of the Mediterranean Sea and the eastern Atlantic Ocean and also in the Black Sea [51,52] Most studies of *Cystoseira* sp. have shown a large variety of secondary metabolites with biological activity including phlorotannins, terpenoids and carbohydrates [53]. *Cystoseira barbata* recovered from the coasts of Tunisia has been proven to contain compounds that show biological activity, including laminarin, which has antioxidant, antibacterial and wound healing properties [54]; fucoxanthin, used as a color enhancer and oxidative stability enhancer of meat products [55]; and polyphenolic-protein-polysaccharide ternary conjugates, which are used as biopreservatives [56]. In the Black Sea, *C. barbata* and *Cystoseira crinita* are the only representatives of *Cystoseira* sp., although a *Cystoseira bosphorica* member has sometimes been reported [51,57]. *C. barbata* is traditionally used in the Black Sea area as functional food [2,58]. Recent studies have been based on ecological interest in the levels of heavy elements in certain parts of the Black Sea coast [59,60,61], as well as the structure of certain metabolites found in *C. barbata* and *C. crinita* [62]. The use of *Cystoseira* spp. biomass as a biosorbent has been shown in one study involving *C. amentacea* var. *stricta* (formerly *C. stricta*) [47]. Dried *C. barbata* biomass from the Turkish coast of the Black Sea has also been investigated as a biosorbent [46]. To the best of our knowledge, the structure of alginate extracted from *C. barbata* and its use as a biosorbent in the form of beads to adsorb heavy metals has never been studied.

The aim of this paper is to characterize alginate extracted from *C. barbata* recovered from the Romanian Black Sea and to prove that this bioactive component presents heavy metal adsorption properties compatible with its use as an adsorbent/biosorbent for heavy metal detoxification. The adsorption properties of the initial seaweed powder were also evaluated.

## 2. Results and Discussion

### 2.1. Extraction and Structural Characterization

In recent years, many studies on the characterization of polysaccharides extracted from brown algae, and more specifically on *Cystoseira* sp. have been conducted and described in the literature [63,64,65]. Nevertheless, alginate extracted from *C. barbata* recovered from the Romanian Black Sea coast has never been investigated from a structural point of view.

**Figure 1:**
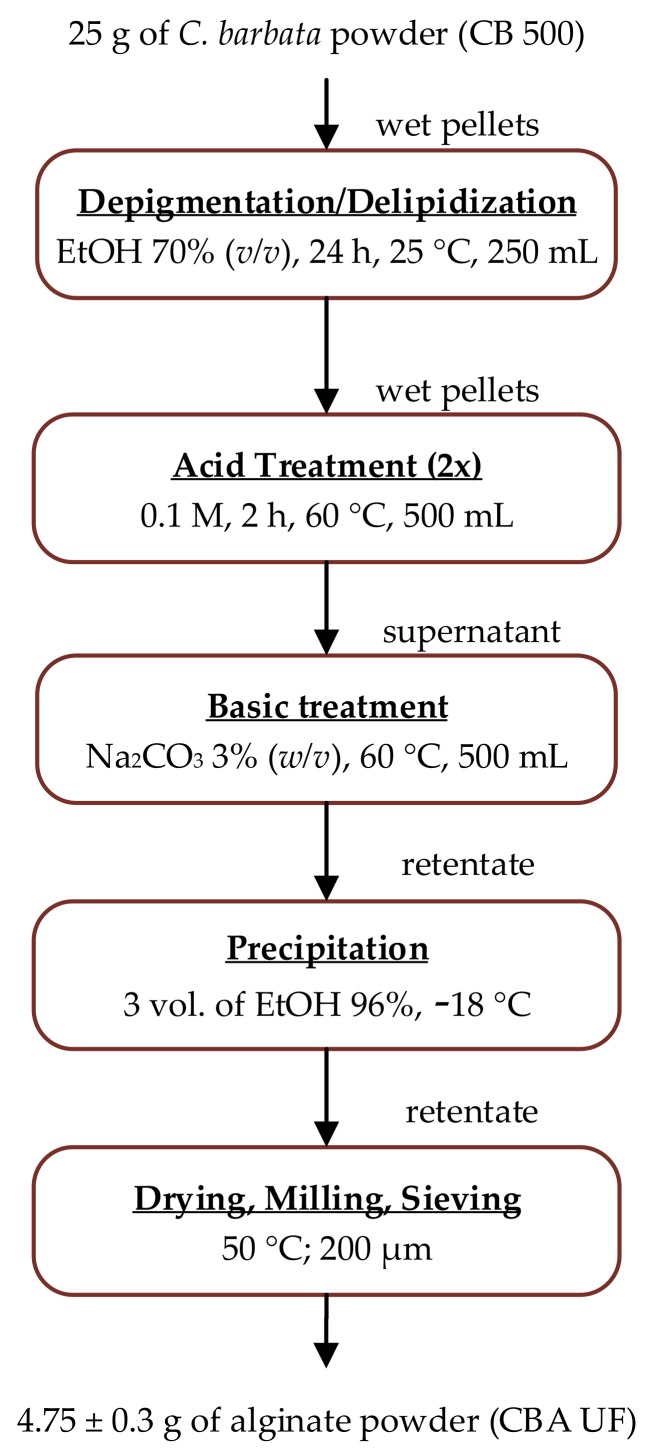
Processing steps for the extraction of alginate from *C. barbata* dry seaweed biomass.

The extraction method (Figure 1) that was employed for the extraction of alginate had a yield of 19 ± 1.5% (*w*/*w*) and relative to the algae dry matter estimated at 95 ± 2%. This method is presented in detail in Section 3.2. The final alginate product is obtained as a fine powder (under 0.2 mm), which is referred to as CBA UF.

### 2.2. HPSEC-MALS

The average molecular weight in mass (M_w_), average molecular weight in number (M_n_) and the intrinsic viscosity of alginate (CBA UF) extracted from *C. barbata* were determined by high performance size-exclusion chromatography equipped with a multi-angle light diffusion detector coupled to a differential refractometer and an in-line viscometer. The recovery rate of the sample was estimated at 90%.

The values of the determined average molecular weights and hydrodynamic radius are reported in Table 1. The M_n_ and M_w_ of alginate (CBA UF) were estimated at 85.2 (±2.7%) kDa and 126.6 (±1.0%) kDa, indicating a low polydispersity index (PDI = M_w_/M_n_ =1.49). These results are similar to those for other alginates obtained from brown algae, and especially from *Cystoseira* sp. such as *Cystoseira sedoides* [66], *Cystoseira compressa* [63,66], *C. crinita* [66]. In fact, our present results for the M_w_ values were very consistent with earlier investigations on alginates from Fucales algae families, for example, *Fucus vesiculosus*, *A. nodosum*, *C. compressa* or *C. sedoides*, which have M_w_ values ranging from around 100 kDa to 200 kDa [66,67]. Nevertheless, compared to other brown algae from *Sargassum* species, where M_w_ values ranged from 300 to 1000 kDa, the M_w_ of alginate fractions (CBA UF) extracted from Romanian *C. barbata* were lower [67,68].

Compared with other alginates from brown algae, and especially from *Cystoseiraceae* species, the PDI value of 1.49 is very close to the alginate from Tunisian *C. compressa* [63]. This PDI value of alginate from Romanian *C. barbata* confirms a good M_w_ polysaccharides distribution, and indicates that there is no depolymerization of polysaccharides during the extraction/purification process steps. Finally, the intrinsic viscosity, which signifies the hydrodynamic volume occupied by the macromolecules in a dilute solution, was estimated for CBA UF at 406 mL/g. This value is lower than the alginate [ƞ] values from *Sargassum* (800–1300 mL/g) brown algae but is close to other intrinsic viscosity values of alginate extracted from *Cystoseiraceae* species [63,64,68]. Notably, a similar [ƞ] was observed with alginate extracted from Tunisian brown algae *C. compressa* [63]. As generally described, these observed differences are related to the origin of the algae and the extraction / purification processes used, which affect molecular weight and intrinsic viscosity.

### 2.3. NMR Analysis

**Figure 2:**
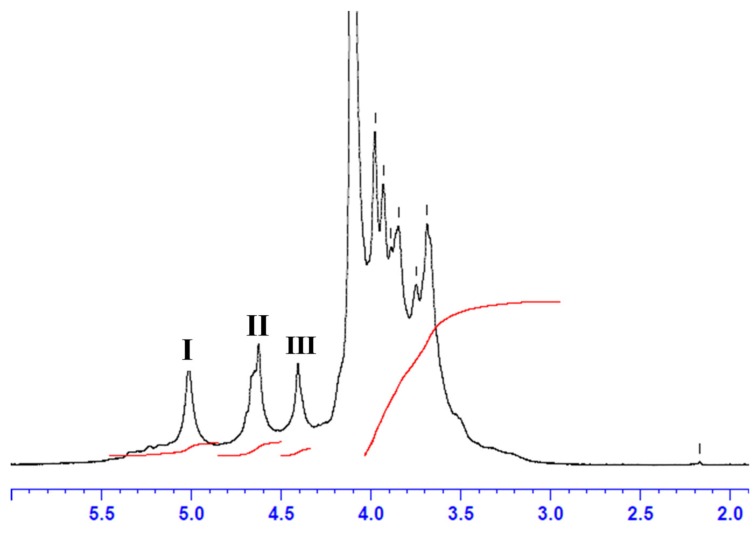
^1^H NMR analysis of CBA UF extracted from *C. barbata* with: signal I = guluronic acid anomeric proton (G-1), signal II = overlap between the mannuronic acid anomeric proton (M-1) and the H-5 of alternating blocks (GM-5), signal III = guluronic acid H-5 position (block GG-5G), G-6 the C-6 from guluronic acid residue and M-6 the C-6 from mannuronic residue.

Alginate extracted from *C. barbata* (CBA UF) was analyzed by ^1^H NMR. As observed in Figure 2, the 1D ^1^H-NMR spectrum showed the specific signal characteristics of the sodium alginate fraction, revealing high purity [69,70].

As well defined in the literature [69,70], ^1^H-NMR analysis identifies the alginate structure with three signals in the anomeric region: Signal I corresponds to the anomeric proton of the guluronic acid residue (G-1), Signal II corresponds to the overlap between the mannuronic acid anomeric proton (M-1) and the H-5 of alternating blocks (GM-5), and Signal III corresponds to proton H-5 guluronic acid from the GG-5G block (G-5). As a general rule, by using the NMR method we could estimate the proportions of each individual block of guluronic and mannuronic acids (F_G_ and F_M_), the homogeneous (F_GG_ and F_MM_) and heterogeneous (F_GM_ and F_MG_) blocks of alginate [70] extracted from *C. barbata* (CBA UF) using the areas (A) of signals I, II and III and the Equations (1), (2) and (3):

(1) $$F_{G}=A_{I}/A_{II}+A\_III$$

(2) $$F_{M}=1-F_{G}$$

(3) $$F_{GG}=A_{III}/A_{II}+A_{III}$$

Regarding the estimation of the block F_GM_, F_MM_ and M/G molar ratio of alginate extracted from *C. barbata* (CBA UF), we used the following equations:

(4) $$F_{GM}=F_{G}-F_{GG}$$

(5) $$F_{MM}=F_{M}-F_{GM}$$

(6) $$M/G=F_{M}/F_{G}$$

Consequently, Equations (4), (5) and (6) allow the complete structural characterization [70] of CBA UF, which is summarized in Table 2.

The frequencies of structural blocks shown in Table 2 provide information about the alginate composition in *C. barbata*. For example, the ratio between mannuronic and guluronic acid gives information about the quality of Ca^2+^ reticulated gels which, in this case, has a strong and rigid quality [71]. This value is generally higher than those reported for other species from *Cystoseira* genus such as *Sirophysalis trinodis* (formerly *C. trinodis*) (0.59), *C. myrica* [68], although it is lower in some cases, *C. compressa* (0.77) [63], *C. humilis* (1.46) [72]. Compared to other species from *Laminaria* or *Sargassum*, this value is higher in some cases (*L. hyperborea*, 0.41 [70]; *Sargassum filipendula*, 0.19 [73]), and lower in other cases (*S. vulgare*, 1.27 [69]; *L. digitata*, 1.12 [74]).

Structural information can be derived by evaluating the parameter η = 1.13 obtained using Equation (7). The value suggests that alginate extracted from *C. barbata* is of an alternate block type since it is greater than 1 [75]. Surprisingly, alginates extracted from other species from the *Cystoseira* genus present η < 1, a feature of alginates that have predominantly homopolymeric blocks [63,68,72].

(7) $$\eta =\frac{F_{GM}}{F_{M}\cdot F_{G}}$$

### 2.4. Kinetics of Adsorption

Sodium alginate beads were obtained as described in Section 3.5. Photos (at 10× magnification) of the beads (not shown here) were taken and analyzed digitally in order to determine the diameter. Twenty beads were analyzed, and the mean diameter was determined to be 4413 ± 134 µm.

Initially, the kinetics of adsorption was studied by contacting sodium alginate beads and dry seaweed powder (<500 µm) with Cu^2+^ and Pb^2+^ solutions at 20 ppm and 74 ppm, respectively (C_i_). [14] Four pairs of substrate/heavy metal were obtained. The instantaneous sorption capacity (q_t_) was determined as defined in Equation (8) where C_t_ is the concentration of heavy metals at time t. This equation is deduced from mass balance. S_m_ represents the value of substrate mass divided by the volume of solution that was used.

(8) $$q_{t}\left(mg/g\right)=\frac{C_{i}-C_{t}}{S_{m}}$$

**Figure 3:**
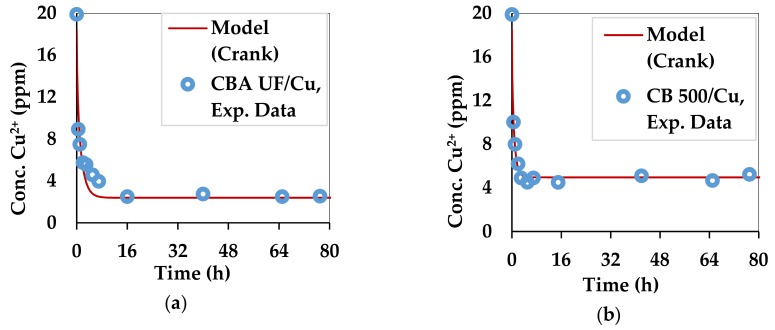
Kinetics of copper adsorption by the two substrates: (**a**) *C. barbata* alginate (CBA UF) and (**b**) *C. barbata* powder (CB 500).

**Figure 4:**
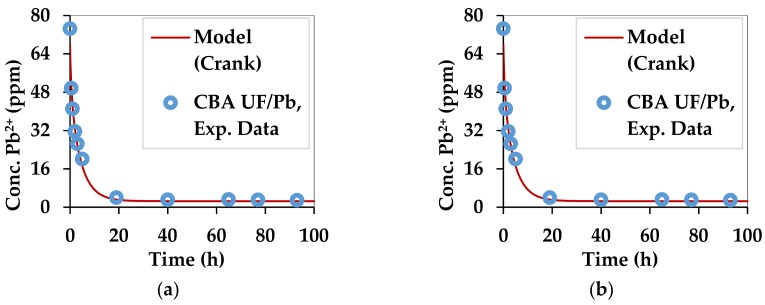
Kinetics of lead adsorption by the two substrates: (**a**) *C. barbata* alginate (CBA UF) and (**b**) *C. barbata* powder (CB 500).

The kinetic curves for copper and lead adsorption of each of the two substrates are shown in Figure 3 and Figure 4.

The diffusion equation represented by Equation (9) [76] was used to simulate the experimental results of adsorption of Pb^2+^ and Cu^2+^ and is based on the diffusion of ions from a solution at a given ion concentration until the solution is solid free or has a constant negligible concentration of ions, which is the case of the present study. α in Equations (9) and (10) represents the ratio between the volume of the liquid and the volume of the solid. In the case of the alginate beads, the volume of solid is represented by the total volume of the beads used. When algal powder is used, the volume of the solute can be approximated by the equivalent volume of water and powder, which is taken up by the powder when swelling. This value is 4.14 times greater than the mass of the used powder. The ratio of a sphere equivalent in volume to the substrate needs to be found for both the alginate and the powder. This term is named a. The effective diffusivity of the solute in the solid is termed D_eff_ (m^2^/s). q_n_ represents the six non-zero roots of Equation 10. q_e_ represents the equilibrium value of the adsorption while q_t_ is the value of the ion adsorption at time t.

(9) $$\frac{q_{t}}{q_{e}}=1-\overset{\infty }{\underset{n=1}{\sum }}\frac{6\alpha \left(\alpha +1\right)\exp\left(-D_{eff}q_{n}{}^{2}t/a^{2}\right)}{9+9\alpha +q_{n}{}^{2}\alpha^{2}}$$

(10) $$\tan q_{n}=\frac{3q_{n}}{3+\propto q_{n}{}^{2}}$$

Figure 3 and Figure 4 compare the experimental and theoretical results from using Equation (9) by regression. The parameters that were optimized are q_e_ and D_eff_. The optimization algorithm, GRG Nonlinear was applied in Excel 2013 by the Solver tool. The Multistart feature was used with a population size of 100, using central derivatives to converge towards the solution. Upper and lower bounds with physical significance were applied for each optimized parameter. The combinations of D_eff_ and q_e_ which best fitted the experimental data for all 4 combinations of substrate and divalent metal ions are summarized in Table 3.

The values of D_eff_ in all cases are similar to the self-diffusivity of Cu^2+^ and Pb^2+^ in water: 0.71 × 10^−9^ m^2^/s and 0.95 × 10^−9^ m^2^/s [77]. This can be explained by the high content of water in the beads and the swollen biomass. Alginate gels are generally nanoporous [6,78], leading to high diffusion rates of small solutes [6]. However, in this case D_eff_ is accelerated by the affinity of divalent metal ions towards the egg-box structure of gels [39] formed by the G fractions in the alginate structure. The Ca^2+^ ions, which occupy this site initially, are replaced by the Pb^2+^ and Cu^2+^ ions, which have a higher affinity [40]. The q_e_ is the maximum adsorption capacity in the given setup, which was not meant to saturate the substrate. The diffusivities obtained for the substrates used in this work are higher than those obtained for materials used in a similar work for Pb^2+^ and Cu^2+^ [79].

### 2.5. Adsorption Isotherms

**Figure 5:**
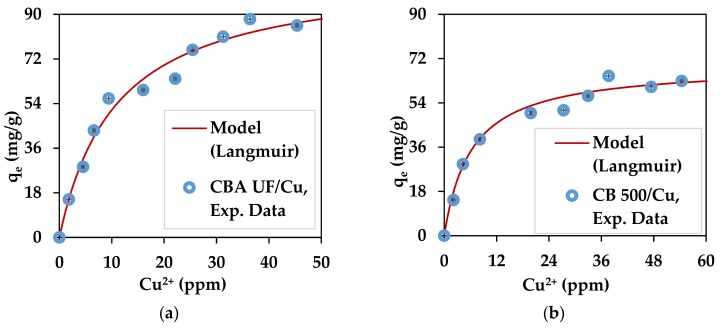
Copper adsorption isotherms for: (**a**) *C. barbata* alginate (CBA UF) and (**b**) *C. barbata* powder (CB500).

**Figure 6:**
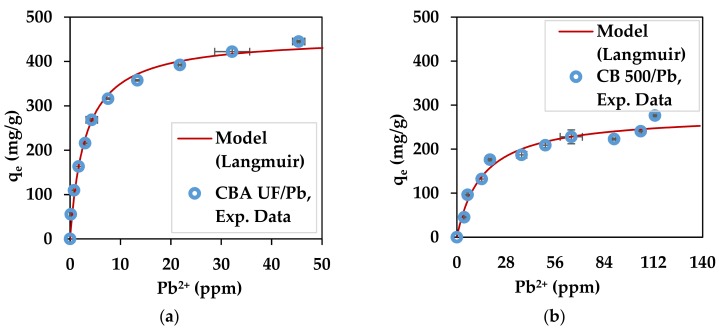
Lead adsorption isotherms for: (**a**) *C. barbata* alginate (CBA) and (**b**) *C. barbata* powder (CB500).

The experimental values for the maximum sorption capacities deduced from the adsorption isotherms are represented in Figure 5 and Figure 6 and are presented in Table 4. Such levels of Pb^2+^ concentration are in the range of contaminated mine waters [80,81].

On the same figures, the Langmuir adsorption model is shown for optimized parameters: q_max_ and K_L_. This model has been used in many studies that involve the adsorption of pollutants such as heavy metals, dyes and phenol [24,82,83]. This model is represented by Equation (11). C_e_ represents the concentration of metal ions still in the solution.

(11) $$q_{e}=\frac{q_{max}K_{L}C_{e}}{1+K_{L}C_{e}}$$

The optimized parameters for each substrate/metal pair are shown in Table 4.

In all cases the capacity of the substrates to adsorb the heavy metals used in this work remains high and reliable because the standard deviation is very low. Interestingly, the seaweed powder without any treatment also had excellent capacity for the adsorption of heavy metals. This is an interesting result because it shows that the brown seaweed recovered from the Romanian Black Sea can be used in its native form for heavy metal adsorption. The values obtained for q_max_ show that the substrates used for both metal ions have good theoretical adsorption capacity according to Langmuir (infinite equilibrium time considered). These values are similar to results obtained in a previous work [79] where for Pb^2+^, alginate beads were found to have a q_max_ of 390.3 mg/g, while brown seaweed biomass (*L. digitata*) was shown to have a q_max_ value of 264.2 mg/g [79]. For Cu^2^,^+^ similar results can be found in the literature with, for example, values of 107.5 mg/g and 74.5 mg/g for alginate and algal biomass (*L. digitata*), respectively [79]. The algal biomass can also be compared to other agricultural wastes that have been investigated as heavy metal adsorbents, such as treated green coconut (*Cocos nucifera*) shells, Pb^2+^: 54.62 mg/g, Cu^2+^: 41.36 mg/g [84]; apricot stone activated carbon, Pb^2+^ 22.85 mg/g, Cu^2+^ 24.21 mg/g [85]; tea waste, Cu^2+^: 48 mg/g [86]; and rose waste biomass, Pb^2+^: 151.51 mg/g [87]. As can be observed, the algal powder has a better adsorption capacity than other dried biomasses. The heavy metals adsorption capacity of *C. barbata* from the Black Sea has been studied before on samples recovered from the Turkish Black Sea Coast [46], where dried *C. barbata* biomass was found to have a maximum sorption capacity of 253 mg/g for lead, which is only slightly lower than the value obtained in this study. Other seaweed from *Cystoseira* sp. have also shown to have heavy metal adsorption properties when used directly as dried biomass. *Cystoseira amentacea* var. *stricta* (formerly *C. stricta*) recovered on the Algerian coast proved to adsorb 64.5 mg/g of lead ions after undergoing several chemical treatments [47].

The good performance of alginate from *C. barbata* can be explained by the low value of the M/G ratio (0.64), which indicates the higher availability of G blocks [40,88]. This could explain the better performance compared to other studies where alginate from *L. digitata* was used [79]. *L. digitata* was shown to have an M/G value of 1.12 [74] or 1.63 [44].

The natural capacity of *C. barbata* to adsorb heavy metals is proven in this study and confirms the high adsorption capacity which was also proven in a previous study [46]. The affinity of brown seaweed for heavy metals can be explained by the presence of alginate, especially the G homogeneous block fractions, which chelate divalent metal ions. This is why alginate extracted from *C. barbata* has an increased capacity.

The good heavy metal absorption characteristics of the edible brown seaweed *C. barbata* and of its major bioactive component, alginic acid, offer a possible explanation regarding the traditional use of this seaweed as functional food. Further studies on biological systems are needed.

Beside its use as nutraceutical/functional food, the biomass of *C. barbata* and/or of its bioactive component, alginic acid, could be used for water treatment to recover heavy metals ions. In this case, this seaweed and the alginate extracted from it have the potential to be used to develop systems such as cartridges, which could facilitate their use in processes under continuous conditions [44]. Also, the behavior during sorption/desorption cycles needs to be determined to prove reusability.

## 3. Materials and Methods

### 3.1. Raw Material and Chemicals

*C. barbata* seaweed was recovered from the Romanian seashore of the Black Sea in the city of Mangalia (GPS coordinates at latitude: N 43° 49.2′, longitude: E 28° 35.4′). The biomass was thoroughly washed with tap water to remove sand particles and epiphytes and dried at room temperature for one week in the dark to prevent any possible degradation associated with sunlight. Afterwards, the dry biomass was milled and sieved at 500 µm to obtain the material referred to as CB 500. The powder recovered under the sieve was used for further manipulation. The chemicals used for this work were analytical grade and were purchased from Sigma-Aldrich (Sigma-Aldrich, Saint-Louis, MO, USA).

### 3.2. Extraction of Alginate

Alginate was extracted by adapting several methods used in the literature [64]. Initially, 25 g of dried seaweed powder underwent a mild depigmentation and defatting in EtOH (70% *v*/*v*, 24 h, 250 mL). The solid was removed by vacuum filtration (Whatman filter paper, 25 µm, Whatman, Maidstone, UK) and added to HCl (0.1 M, 2 h, 60 °C, 500 mL). After vacuum filtration, this operation was repeated for the recovered wet pellets. The excess acid was washed away with distilled water before extracting the alginate in a Na_2_CO_3_ solution (3% (*w*/*v*), 2 h, 60 °C). The sodium alginate extract was left to cool before separating it from the solid waste by centrifugation (15,000 g, 30 min, 4 °C). After neutralization with dilute HCl, the extract was treated by ultrafiltration on a Vivaflow 200 crossflow cassette module (100 kDa, polyethersulfone, Sartorius, Göttingen, Germany) fitted with a peristaltic pump. Following the treatment in diafiltration mode with 5 diavolumes, alginate remained in solution in the retentate while most of contaminants were removed in the permeate. This procedure was followed by a concentration process, which reduced the volume of the retentate 3 times before precipitating the alginate by adding 3 volumes of EtOH (96%, −18 °C). The alginate pellets were dehydrated twice with 20 mL of acetone at −18 °C. The pellets were then dried at 50 °C, milled and sieved (0.2 mm) to obtain a fine powder called CBA UF.

### 3.3. NMR Analysis

The sample was prepared and analyzed under conditions described in the literature [63]. CBA UF was dissolved in D_2_O (99.9% D) at a concentration of 50 g/L. After dissolution the sample was freeze-dried resulting in CBA UF alginate with exchangeable protons replaced by deuterium. In total, this operation was done 3 times. Before analysis, the CBA UF lyophilized sample was dissolved again in D_2_O (99.9% D) at a concentration of 40 g/L. NMR spectra were obtained at 80 °C using a 400 MHz Bruker Avance spectrometer (Bruker, Billerica, MA, USA), equipped with a BBFO probe. A spectral width of 3000 Hz was used for acquiring the data obtained under the following acquisition parameters: acquisition mode = 2 s, pulse 90° = 8 μsec, scans = 64, recovery = 5 s (for a complete return after the 90° pulse).

### 3.4. HPSEC-MALS

High pressure size exclusion chromatography (HPSEC) was used to determine the number average molar mass, (M_n_), the mass average molar mass (M_w_), intrinsic viscosity ([η]), hydrodynamic radius (R_h_) and gyration radius (R_g_) for CBA UF. Three detectors were used in parallel, and were coupled to the chromatograph: multi-angle light scattering (MALS, DAWN-EOS from Wyatt Technology Corp., Santa Barbara, CA, USA) with a Ga-As laser (690 nm) and a K5 cell (50 µL) (HELEOS II Wyatt Technology Corp., USA), a viscosimeter (Viscostar II, Wyatt Technology Corp., USA) and refractive index detector (RID, RID10A Shimadzu, Kyoto, Japan). One OHPAK SB-G, two OHPAK SB 804 and 806 HQ columns were used in series for the HPSEC line (Shodex Showa Denko K.K., Tokyo, Japan). The system was eluted with LiNO_3_ 0.1 M, filtered using a 0.1 µm unit (Millipore, Merck Group, Darmstadt, Germany) and degassed (DGU-20A3 Shimadzu, Kyoto, Japan). The flow rate was set at a value of 0.5 mL/min (LC10Ai Shimadzu, Kyoto, Japan). The sample was diluted to 1 mg/mL in LiNO_3_ 0.1M under stirring for 24h, filtered (0.45 µm, Millipore) before 500 µL was placed onto the analytical line of the instrument with an automatic injector (SIL-20A Shimadzu, Kyoto, Japan).

### 3.5. Preparation of Alginate Beads

CBA UF was dissolved in MilliQ water to obtain a solution with a concentration of 3% (*w*/*v*). The solution was then pumped through a capillary (d = 1 mm) with its other end above a 0.5 M CaCl_2_ solution under stirring. Alginate beads are formed drop by drop as they contact the calcium solution. They were stored for 12 h at 4 °C in a 0.5 M CaCl_2_ solution. A digital camera (Euromex CMEX-5000, Arnhem, The Netherlands) was used to take photos of the alginate beads. The diameter of the beads was measured using the ImageFocus software.

### 3.6. Adsorption Kinetics

The initial conditions for the kinetic experiments were adapted from the literature [79] and are shown in Table 5. The substrate mass represents the quantity of alginate used to produce the corresponding number of beads used or the mass of dried powder added. The initial concentration of heavy metal ions is also given in Table 5 and was obtained from PbCl_2_ and CuSO_4_. The corresponding quantity of substrate was added under stirring. The kinetics experiments were run for 100 h in order to achieve equilibrium. The concentration of metal ions was determined using MP-AES (microwave plasma-atomic emission spectrometry, Agilent 4200, Santa Clara, CA, USA).

### 3.7. Adsorption Isotherms

Following the adsorption experiments, it was observed that the equilibrium was well reached after ~65 h. This value is used as the time necessary to achieve equilibrium in the adsorption isotherm experiments. Ten solutions of 50 mL each were obtained for each substrate/heavy metal pair with a concentration of heavy metal ions varying from 22.3 to 223 ppm for Pb^2+^ and from 8 to 79.6 ppm for Cu^2+^(C_i_). In this case, the mass of alginate contained in the beads with respect to the heavy metal solution (S_m_) was fixed at 0.4 g/L of solution. The corresponding mass of substrate was added to the heavy metal solutions. The solutions are kept under agitation using a plate shaker until equilibrium was reached. Afterwards, the final concentration of heavy metals was determined by MP-AES.

## Figures and Tables

**Table 1:** Characterization of alginate (CBA UF) extracted from *C. barbata* collected from the Romanian Black Sea.

	Mn (kDa)	Mw (kDa)	PDI	R_h_(w) (nm)	[ƞ] (mL/g)
CBA UF	85.2	126.6	1.49	19.2	406.2

Mn—average molecular weight in number; Mw—Average molecular weight in mass (Mw); PDI—polydispersity index; R_h_(w)—hydrodynamic radius; [ƞ]—intrinsic viscosity.

**Table 2:** Structural characterization of alginate (CBA UF) extracted from *C. barbata* from Romanian Black Sea.

Fraction	F_G_^1^	F_M_^2^	F_GG_^3^	F_GM_ or F_MG_^4^	F_MM_^5^	M/G ^6^
CBA UF	0.61	0.39	0.34	0.27	0.12	0.64

^1^ F_G_—fraction of individual blocks of guluronic acid units; ^2^ F_M_—fraction of individual blocks of mannuronic acid units; ^3^ F_GG_—fraction of homogeneous block of guluronic acid; ^4^ F_GM_ or F_MG_—fraction of heterogeneous blocks of alternating mannuronic and guluronic acids; ^5^ F_MM_—fraction of homogeneous block of mannuronic acid; ^6^ M/G—ratio between F_M_ and F_G_.

**Table 3:** Crank diffusion model for a sphere. Model coefficients for 4 pairs of substrate / heavy metal.

Substrate/Metal Ion	D_eff_ × 10^−9^(m^2^/s)	q_e_ (mg/g)
CBA UF/Pb^2+^	0.85	359.8
CBA UF/Cu^2+^	3.98	43.8
CB 500/Pb^2+^	1.39	172
CB 500/Cu^2+^	1.79	37.3

D_eff_—the effective diffusivity of the solute in the solid; q_e_—the equilibrium value of the adsorption.

**Table 4:** Adsorption isotherms - model parameters (Langmuir).

Substrate/Metal Ion	q_max_ (mg/g)	K_L_ (mg/L)	U_t_ (mmol/g)
CBA UF/Pb^2+^	454 ± 4.7	0.32 ± 0.04	0.77
CB 500/Pb^2+^	279.2 ± 7.5	0.069 ± 0.005	0.15
CBA UF/Cu^2+^	107.3 ± 1.7	0.092 ± 0.005	0.77
CB 500/Cu^2+^	69.3 ± 2	0.16 ± 0.03	0.15

q_max_—maximum theoretical adsorption capacity according to Langmuir, K_L_—Langmuir constant, U_t_—theoretical egg-box sites per mass of substrate.

**Table 5:** Initial conditions for adsorption kinetics experiments.

Substrate/Heavy Metal Pair	Substrate Mass/Solution Volume (g/L)	Initial Concentration of Heavy Metal Ions (ppm)	Heavy Metal Ions/Dry Substrate (*w*/*w*)
CBA UF/Pb^2+^	0.2	74	0.37
CBA UF/Cu^2+^	0.4	20	0.05
CB500/Pb^2+^	0.4	74	0.185
CB500/Cu^2+^	0.4	20	0.05
